# Survival outcome of cervical cancer patients treated by image-guided brachytherapy: a ‘real world’ single center experience in Thailand from 2008 to 2018

**DOI:** 10.1093/jrr/rrac025

**Published:** 2022-06-19

**Authors:** Ekkasit Tharavichitkul, Bongkot Jia-Mahasap, Pooriwat Muangwong, Somvilai Chakrabandhu, Pitchayaponne Klunklin, Wimrak Onchan, Damrongsak Tippanya, Wannapa Nobnop, Anirut Watcharawipha, Kittikun Kittidachanan, Ravan M Galalae, Imjai Chitapanarux

**Affiliations:** The Division of Radiation Oncology, Department of Radiology, Faculty of Medicine, Chiang Mai University, Chiang Mai 50200, Thailand; Northern Thailand Radiation Oncology Group, Faculty of Medicine, Chiang Mai University, Chiang Mai 50200, Thailand; The Division of Radiation Oncology, Department of Radiology, Faculty of Medicine, Chiang Mai University, Chiang Mai 50200, Thailand; Northern Thailand Radiation Oncology Group, Faculty of Medicine, Chiang Mai University, Chiang Mai 50200, Thailand; The Division of Radiation Oncology, Department of Radiology, Faculty of Medicine, Chiang Mai University, Chiang Mai 50200, Thailand; Northern Thailand Radiation Oncology Group, Faculty of Medicine, Chiang Mai University, Chiang Mai 50200, Thailand; The Division of Radiation Oncology, Department of Radiology, Faculty of Medicine, Chiang Mai University, Chiang Mai 50200, Thailand; Northern Thailand Radiation Oncology Group, Faculty of Medicine, Chiang Mai University, Chiang Mai 50200, Thailand; The Division of Radiation Oncology, Department of Radiology, Faculty of Medicine, Chiang Mai University, Chiang Mai 50200, Thailand; Northern Thailand Radiation Oncology Group, Faculty of Medicine, Chiang Mai University, Chiang Mai 50200, Thailand; The Division of Radiation Oncology, Department of Radiology, Faculty of Medicine, Chiang Mai University, Chiang Mai 50200, Thailand; Northern Thailand Radiation Oncology Group, Faculty of Medicine, Chiang Mai University, Chiang Mai 50200, Thailand; The Division of Radiation Oncology, Department of Radiology, Faculty of Medicine, Chiang Mai University, Chiang Mai 50200, Thailand; Northern Thailand Radiation Oncology Group, Faculty of Medicine, Chiang Mai University, Chiang Mai 50200, Thailand; The Division of Radiation Oncology, Department of Radiology, Faculty of Medicine, Chiang Mai University, Chiang Mai 50200, Thailand; Northern Thailand Radiation Oncology Group, Faculty of Medicine, Chiang Mai University, Chiang Mai 50200, Thailand; The Division of Radiation Oncology, Department of Radiology, Faculty of Medicine, Chiang Mai University, Chiang Mai 50200, Thailand; Northern Thailand Radiation Oncology Group, Faculty of Medicine, Chiang Mai University, Chiang Mai 50200, Thailand; The Division of Radiation Oncology, Department of Radiology, Faculty of Medicine, Chiang Mai University, Chiang Mai 50200, Thailand; Northern Thailand Radiation Oncology Group, Faculty of Medicine, Chiang Mai University, Chiang Mai 50200, Thailand; MedAustron Ion Therapy Center, Wiener Neustadt 2700, Austria; Faculty of Medicine, Christian-Albrechts-University, Kiel 24118, Germany; The Division of Radiation Oncology, Department of Radiology, Faculty of Medicine, Chiang Mai University, Chiang Mai 50200, Thailand; Northern Thailand Radiation Oncology Group, Faculty of Medicine, Chiang Mai University, Chiang Mai 50200, Thailand

**Keywords:** survival, outcome, cervical cancer, image-guided brachytherapy (IGBT)

## Abstract

The objective of our study was to evaluate the survival outcome of cervical cancer patients treated using image-guided brachytherapy (IGBT). From 2008 to 2018, 341 patients with cervical cancer were treated by radical radiotherapy. IGBT (by computed tomography [CT] or transabdominal ultrasound [TAUS]) was used to treat all of these patients. The characteristic data and patient status after treatment were recorded. All data were evaluated for survival outcome analysis. From a total of 341 patients, 295 patients were analyzed and 46 patients were excluded due to data missing in the survival outcomes. At the median follow-up time of 48 months (IQR 30–80 months), The 4-year local control, progression-free survival and overall survival rates were 89.5%, 74.9% and 69.1%, respectively. For overall survival, the size (> 5 cm), pathology (non-SCCA), stage (stage III–IV by FIGO 2009), lymph node (LN) (presented) and overall treatment time (OTT) (> 56 days) showed statistical significance in univariate analysis while non-SCCA pathology, advanced stage, presented LN and longer OTT showed statistical significance in multivariate analysis. In conclusion, our analysis reports a 4-year overall survival rate of 69.1%. Non-SCCA pathology, advanced stage disease, LN presence and longer OTT showed worse prognostic factors in multivariate analysis.

## INTRODUCTION

Carcinoma of cervix uteri is one of the most common female cancers in Northern Thailand [1]. Radiotherapy (external beam radiotherapy [EBRT] plus brachytherapy [BT]) with platinum-based chemotherapy is the standard treatment for locally advanced disease [2]. After developments in magnetic resonance imaging (MRI) in BT, the concepts of volume-based treatment have been utilized in routine practice since 2005 using concepts of the Groupe Européen de Curiethérapie and the European Society for Radiotherapy & Oncology (GEC-ESTRO) recommendations [3, 4]. Many clinical studies of image-guided brachytherapy (IGBT) using MRI-guided BT have been published and the latest publication of the EMBRACE-I study showed a 5-year local control and survival rate of 92% and 74%, respectively [5–9]. Computed tomography (CT)-IGBT has also been also used in many institutes. Although CT yields poorer image quality than MRI in overestimation, CT is more easily accessible and can be adapted to reduce normal tissue dose [10]. To date, CT-based guidelines have been published to support this approach [11–13]. Many studies using CT-based BT have been published to support the use of CT in BT practice [14–17]. Transabdominal ultrasound (TAUS) was developed in BT by van Dyk *et al.* and published clinical studies showed promising results in terms of local control and survival [18–21].

Our Institute has transformed our brachytherapy service from 2D to 3D brachytherapy in our division since 2008 with the cooperation of the University Cooperation platform with Christian-Albrechts-University, Kiel and Medical University of Vienna [22]. From 2008 to 2018, we developed two research projects in different proposal. CT-BT project was our forward project to move our department from point-based planning to volume-based planning. During the transformation, due to our workload, we could not transform all of our patients to use volume-based planning at that time. So, at that time, we looked for the technique to support the adaptive planning for point-based planning and we found that TAUS-BT was the suitable one. So, TAUS-BT project was our supported project to improve our 2D by adaptively point-based planning during transformation process. After 10 years, we fully changed to CT-based BT in 2019. We have reported our experiences in CT and TAUS in international publications [16, 17, 20, 21, 23].

After 10 years of implementation (2008–2018), evaluation of the survival outcome is one of our objectives. In this study, we evaluate the survival outcomes of IGBT (by CT and TAUS) in our Institute.

## MATERIALS AND METHODS

This retrospective study evaluates the survival outcomes of cervical cancer patients treated by IGBT in our Institute. This study was approved by the institutional review board of Faculty of Medicine, Chiang Mai University with the study code of RAD-2564-08287.

**Fig. 1:**
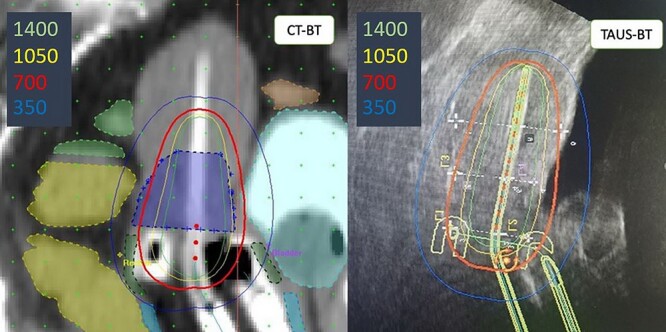
TAUS and CT for BT.

From 2008 to 2018, 341 patients with cervical cancer were treated by IGBT. There were 176 patients treated by CT-based BT while 165 patients were treated by TAUS-based BT. In our hospital, 3D-BT began in 2008 with CT-based BT as a research project. Later, another project to improve the quality of 2D planning by TAUS-guided BT as 2.5D planning was initiated in 2012. All patients received external beam radiotherapy from 45 to 50.4 Gy in 23–28 fractions plus four fractions of IGBT. In presented macroscopic lymph node (LN), the boost therapy to 56–60 Gy was applied as indicated. Extended field radiotherapy (pelvic plus paraaortic fields) was used for paraaortic LN involvement. Conventional, three-dimensional conformal, or intensity-modulated radiation therapy were utilized in our patients as indicated. Weekly platinum-based regimen (cisplatin; 40 mg/m2 or carboplatin; AUC2) was prescribed concurrently during external beam radiotherapy. For BT, all patients received IGBT by CT- or TAUS-based planning techniques (Fig. 1). Pre-BT MRI did not perform in our cohort. To compensate pre-BT MRI, per vaginal examination and TAUS were utilized. Hybrid intracavitary and interstitial (IC/IS) technique was indicated in case of big or poor geometry targets (whole cervix and macroscopic tumor at BT). For pain control, oral Tylenol with codeine was prescribed for the IC approach. In patients who need IC/IS technique, intravenous pethidine/valium or spinal anesthesia will be used in our practice. The concept of dose prescriptions and cumulative doses in both techniques has been described in previous publications [16, 17, 20, 21]. For our planning aims, we keep the cumulative dose (EBRT plus BT in EQD2) to our target (cervix + macroscopic tumor extension) to be at least 80 Gy, < 90 Gy for bladder and < 75 Gy for rectum. For CT-based BT, the target was the D90 of high-risk clinical target volume (HR-CTV). For TAUS-based BT, the target was generated from the eight cervix reference points measured by TAUS.

All characteristic data (age, stage, histology, chemotherapy, cumulative dose in EQD2) were recorded. All patients were traced to the cancer registry unit to evaluate their updated status (whether dead or alive). The survival data (from start of treatment to death) were evaluated.

In statistical analysis, descriptive statistics were used to evaluate characteristic data. A Kaplan–Meier curve and a log-rank test were utilized to evaluate the overall survival rate. Univariate and multivariate analyses were performed to evaluate the prognostic factor. IBM SPSS version 22 (SPSS Inc., Chicago, Illinois, USA) was used for evaluation.

## RESULTS

**Fig. 2:**
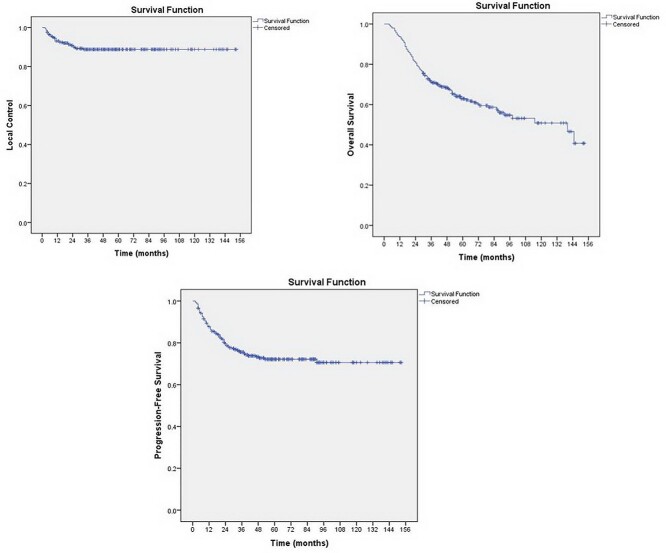
Kaplan–Meier curve of local control, disease-free survival and overall survival.

From 341 patients, 46 patients were excluded because their data were not recorded in the cancer registry. Consequently, 295 patients were used for the evaluation. (Fig. 2) Stage IIB (as FIGO 2009) was the most common with 51.9%. Squamous cell carcinoma (SCCA) was the most common pathology (85.1%). The median age was 57 years and 80% of patients were ≤ 65 years old. One-hundred seventy-nine patients (60.7%) were treated by conventional radiotherapy. Fifty-two patients (17.6%) had macroscopic LN and 18 patients (6.1%) had para-aortic LN involvement. Two-hundred fifteen patients (72.9%) received weekly cisplatin as concurrent chemotherapy and 198 patients (67.1%) received at least four cycles of chemotherapy. The median cumulative dose to the target was 84.8 Gy in EQD2. Twenty-seven patients (9.2%) were treated by hybrid IC/IS technique. Patient characteristic data are shown in Table 1.

**Fig. 3:**
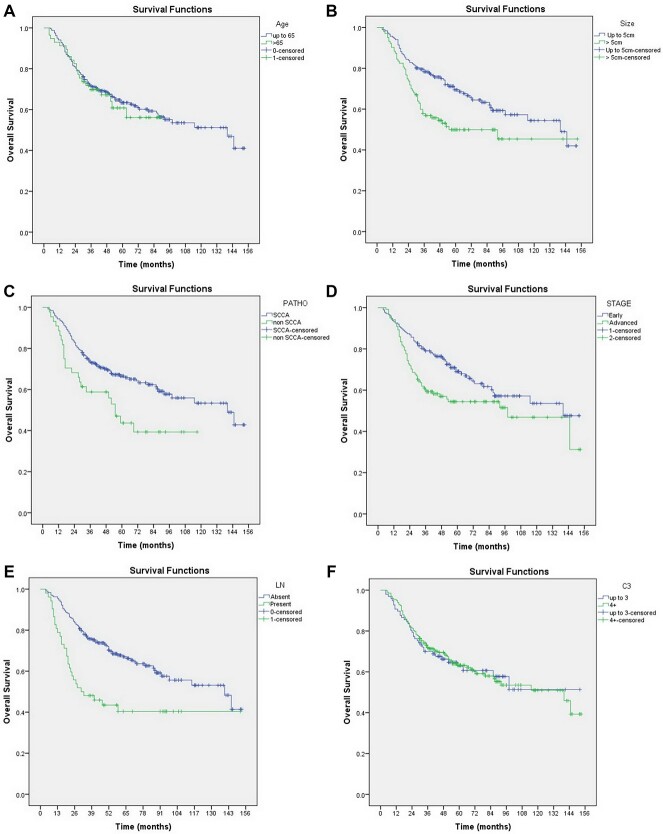

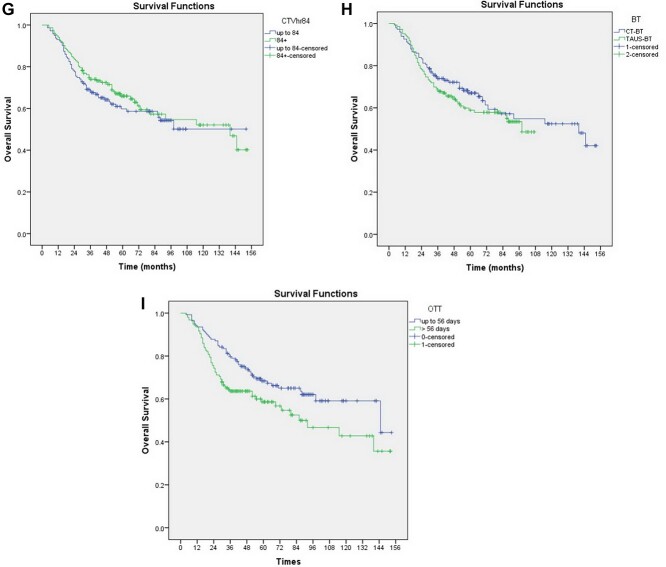
Kaplan–Meier curves of overall survival according to: a) age, b) size, c) pathology, d) stage, e) LN status, f) chemotherapy cycles, g) cumulative dose to target, h) BT technique, and i) OTT.

**Table 1:** Patient characteristic data

Parameters	Details
Age (median; IQR)	57 years (50–63 years)
Size (median; IQR) Up to 5 cm More than 5 cm	5 cm (4–6 cm) 186 (85.1%) 103 (34.9%)
Pathology Squamous cell carcinoma Adenocarcinoma Others	251 (85.1%) 40 (13.6%) 4 (1.4%)
Stage (FIGO 2009) I II III IV	10 (3.4%) 164 (55.6%) 114 (38.7%) 7 (2.4%)
Macroscopic LN No Pelvic LN Paraaortic LN	243 (82.4%) 34 (11.5%) 18 (6.1%)
IGBT CT-based TAUS-based	149 (50.5%) 146 (49.5%)
Cumulative Dose in EQD2 to the target (median; IQR)	84.8Gy (82.2–89.7 Gy)
Overall treatment time (median; IQR)	56 days (50–64 days)

**Table 2:** Uni-variate analyses

Variables	n	4-year local control rate (%)	*P*-value^*^	4-year overall survival rate (%)	*P*-value^*^
Age (years) up to 65 years More than 65 years	238 57	89.1 91.2	0.666	69.3 68.4	0.72
Size (cm) Up to 5 cm More than 5 cm	186 103	91.4 86.4	0.134	**76.3** **55.3**	**0.004**
Pathology SCCA Non-SCCA	251 40	**91.6** **77.3**	**0.002**	**70.9** **59.1**	**0.007**
FIGO Stage Stage I–II (early) Stage III–IV (advanced)	174 121	**93.1** **84.3**	**0.013**	**77.0** **57.8**	**0.015**
LN Absent Present	243 52	90.5 84.6	0.09	**74.5** **44.2**	**<0.001**
Cycles of chemotherapy Less than 4 cycles ≥ 4 cycles	97 198	89.7 89.3	0.96	67 70.2	0.914
Cumulative dose to target Up to 84 Gy > 84 Gy	**143** **152**	**85.3** **93.4**	**0.022**	65.0 73.0	0.386
BT CT TAUS	149 146	90.6 88.4	0.513	73.2 65.1	0.31
OTT Up to 56 days More than 56 days	139 156	**94.2** **85.3**	**0.010**	**74.8** **64.1**	**0.017**

Note: BT = brachytherapy, CA = carcinoma, CT = computed tomography, FIGO = The International Federation of Gynecology and Obstetrics, LN = lymph node, OTT = overall treatment time, SCCA = squamous cell carcinoma, TAUS = transabdominal ultrasound; ^*^ by log-rank test

At the median follow-up time of 48 months (IQR 30–80 months), The 4-year local control, progression-free survival and overall survival rates were 89.5%, 74.9% and 69.1%, respectively. Figure 2 shows the Kaplan–Meier curve of these data. For late toxicity, five patients (1.7%) developed ≥ grade 2 cystitis and 12 patients (4.1%) developed ≥ grade 2 proctitis.

In local control, univariate analysis of age (up to 65 years vs > 65 years), size (up to 5 cm vs > 5 cm), pathology (SCCA vs non-SCCA), FIGO stage (stage I–II vs stage III–IV), LN (absent vs present), chemotherapy cycles (up to 3 cycles vs ≥ 4 cycles), cumulative dose to target (up to 84 Gy vs > 84 Gy), BT (CT vs TAUS) and overall treatment time (OTT; up to 56 days vs > 56 days) were evaluated. The results showed statistical significance in pathology, stage, cumulative dose to target and OTT with non-significant trends in LN. Details of univariate analysis are shown in Table 2. When we evaluated in terms of multivariate-analysis, Cox proportional regression analysis revealed a significant correlation in local control to advanced stage, non-SCCA histology, cumulative dose > 84 Gy and OTT. All evaluations are shown in Table 3.

**Table 3:** Multi-variate analyses

Factor	Local control			Overall survival		
	HR	95%CI	*P*-value	HR	95% CI	*P*-value
Age > 65	0.727	0.242–2.184	0.571	1.374	0.831–2.272	0.216
Size > 5 cm	1.203	0.558–2.594	0.637	1.461	0.989–2.158	0.057
**Non-SCCA**	**3.986**	**1.533–10.365**	**0.005**	**2.414**	**1.440–4.046**	**0.001**
**FIGO stage III–IV**	**2.330**	**1.067–5.088**	**0.034**	**1.606**	**1.078–2.393**	**0.020**
Presented LN	1.675	0.692–4.050	0.253	**1.886**	**1.204–2.952**	**0.006**
≥ 4 Cycles of chemotherapy	0.893	0.346–2.306	0.815	0.942	0.585–1.516	0.806
**Cumulative dose to target > 84Gy**	**0.33**	**0.131–0.832**	**0.019**	0.910	0.555–1.493	0.710
BT (CT vs TAUS)	0.821	0.339–1.985	0.661	1.033	0.637–1.676	0.895
**OTT > 56 days**	**2.520**	**1.063–5.973**	**0.036**	**1.557**	**1.049–2.311**	**0.028**

Note: BT = brachytherapy, CT = computed tomography, FIGO = The International Federation of Gynecology and Obstetrics, HR = hazard ratio, LN = lymph node, OTT = overall treatment time, SCCA = squamous cell carcinoma, TAUS = transabdominal ultrasound

In overall survival, the same nine parameters were evaluated. In terms of univariate analysis, the results showed statistical significance in size, pathology, stage, LN and OTT. Details of univariate analysis are shown in Table 2. The Kaplan–Meier curve of age, size, pathology, stage, LN status, chemotherapy cycles, cumulative dose to target, BT and OTT were showed in Fig. 3a–3i. When we evaluated in terms of multivariate-analysis, Cox proportional regression analysis revealed a significant correlation in overall survival to non-SCCA histology, advanced stage, presented LN and longer OTT. All evaluations are shown in Table 3.

## DISCUSSION

Nowadays, radiotherapy for cervical cancer has changed a lot in the technology of treatment, especially in BT. After the first clinical study was published in 2007 by Pötter *et al.* [5]. The use of IGBT has developed rapidly using MRI-, CT- or ultrasound-based planning [6, 9, 14, 19, 24]. The latest publications of the EMBRACE-I study showed promising results of MRI-guided BT with a 5-year local control and overall survival rates of 92% and 74%, respectively [8]. This result showed an improvement from the previous report of the retro-EMBRACE study that showed 5-year overall survival rate of 65% [7]. From the literature, the survival rate for cervical cancer treated by IGBT ranged from 58 to 86% over 3–5 years. (Table 4).

**Table 4:** Selected studies of IGBT for cervical cancer

**Study**	**n**	**Image**	**Local control rate (%)**	**Survival rate (%)**
Pötter *et al.* [8]	1341	MRI	92% (5 yr)	74% (5 yr)
Möller *et al.* [25]	138	MRI/CT	94.2% (5 yr)	65% (5 yr)
Sturdza *et al.* [7]	731	MRI/CT	89%(5 yr)	65% (5 yr)
Narayan *et al.* [19]	292	TAUS	87.5% (5 yr)	65% (5 yr)
Castelnau-Marchand *et al.* [26]	225	MRI/CT	86.4% (3 yr)	76.1% (3 yr)
Lindegaard *et al.* [24]	140	MRI	91%(3 yr)	79% (3 yr)
Pötter *et al.* [6]	156	MRI	95%(3 yr)	68% (3 yr)
Pötter *et al.* [5]	145	MRI	85%(3 yr)	58% (3 yr)
Ribeiro *et al.* [27]	170	MRI/CT	96%(3 yr)	65% (5 yr)
Ohno *et al.* [14]	80	CT	94% (5 yr)	86% (5 yr)
Our study	295	CT/TAUS	89.5% (4 yr)	69.1% (4 yr)

Note: CT = computed tomography, MRI = magnetic resonance imaging, TAUS = transabdominal ultrasound, yr = year

Our study reports experiences of IGBT by CT or TAUS in our Institute from 2008 to 2018. For our reported data, the 4-year local control, progression-free survival and overall survival rates were 89.5%, 74.9% and 69.1%, respectively. For local control, our report were close to the results from retro-EMBRACE study (89%) and Narayan *et al.* (87.5%) but inferior to EMBRACE-I study (92%) [7, 19]. For overall survival, our results are still close to the results from the retro-EMBRACE study (65%), Möller *et al.* (65%), Ribeiro *et al.* (65%) and Narayan *et al.* (65%) but show inferior results to the EMBRACE-I study (74%) [7, 8, 19, 25, 27].

A possible reason of different results from EMBRACE I study might be that the percentage of stage III patients in the EMBRACE-I study was 15.2% in comparison to 38.7% in our study [8]. However, in our cohort, only FIGO 2009 was evaluated. Additionally, 43% of patients in the EMBRACE-I study used combined IC/IS BT to get dose escalation [8]. From our analysis, 9.2% (27 patients) of our patients were treated by hybrid IC/IS technique at that time. Although the utilization of IC/IS technique in our study was still lower than retro-EMBRACE study (23%), our study showed approximate results in terms of local control and overall survival [7]. After installation of CT in BT unit, we aim that the use of IC/IS technique will increase and this will improve our results in the future.

In our study, nine prognostic parameters (age, size, pathology, stage, presented LN, chemotherapy cycles, cumulative dose to the target, technique of IGBT and OTT) were evaluated in univariate and multivariate analyses. When we evaluated local control, in univariate analysis, our results showed statistical significances in pathology (non-SCCA) stage (III–IV), cumulative dose to target (> 84 Gy) and OTT (> 56 days) with trends in LN (present) **(**Table 2**)** For multivariate-analysis, Cox proportional regression analysis revealed a significant correlation in local control to non-SCCA histology, advanced stage, cumulative dose > 84 Gy and OTT > 56 days (Table 3). In overall survival, size (> 5 cm), non-SCCA histology, advanced stage (III–IV), LN status (presented LN) and OTT (> 56 days) showed statistical significance in univariate analysis (Table 2). When we evaluated using multivariate analysis, non-SCCA histology, advanced stage, presented LN and longer OTT than 56 days were of statistical significance (Table 3). Our analysis is similar to that of Möller *et al.* which showed that FIGO stage and LN metastases were prognostic factors for cancer-specific survival rates [25].

When we focused on SCCA group (251 patients), the 4-year local control, disease-free survival and overall survival rates were 91.6%, 78.1% and 70.9%, respectively. For overall survival, presented LN showed a significant correlation in overall survival in our analysis (*P* = 0.045; HR 1.544; 95% CI = 1.011–2.787) while no statistical significance in size (*P* = 0.055; HR 1.544; 95% CI = 0.991–2.404) and clinical stage (*P* = 0.158; HR 1.371; 95% CI = 0.885–2.124) was observed.

From our analysis, non-SCCA histology, advanced stage and presented LN were the factors which patients presented before treatment, in contrast to cumulative dose to the target and OTT that we can manage. According to the latest publication by Korenaga *et al.*, completing chemoradiotherapy (standard of care) in the recommended 8 weeks was associated with a superior overall survival in cervical cancer [28]. Consequently, to keep the OTT to be as less as possible is very important to achieve favorable results. Previously, one fraction per week was routinely used in our practice and it caused some patients who had treatment break during EBRT got high tendency to treat longer than 8 weeks. Nowadays, twice-a-week schedule is currently utilized in our hospital and we expect that this will improve our results in the future.

Nonetheless, workload is still important obstacle of twice-a-week schedule. For the cumulative dose to target, cumulative dose (> 84 Gy in EQD2) showed statistical significance in local control in our analysis (*P* = 0.019) but, unfortunately, not in overall survival (*P* = 0.710). The plan of our practice to increase cumulative dose to > 84 Gy by hybrid IC/IS technique was designed after installation of CT in the BT unit.

For the technique of BT, no difference in techniques of IGBT (CT vs TAUS) was observed in terms of local control and survival. However, the trends of BT for cervical cancer shift to hybrid IC/IS technique according to the retro-EMBRACE and EMBRACE I studies [7, 8]. Hybrid IC/IS utilization should increase in practice and the further study of CT-based IGBT vs TAUS-based IGBT in hybrid IC/IS approach is interesting. Nevertheless, the planning system for TAUS-based BT in hybrid approaches is during development [29].

The major limitations of the study are as follows. First, it was a retrospective analysis performed over a relatively long period of time (10 years). Second, short median follow-up time was observed (48 months) because some patients were lost to follow-up at our Institution, especially in this COVID-19 era. However, the strength of the present study is that our cohort contains cervical cancer patients who were treated by IGBT from a single institution and that data could be traced in the cancer registry database. The results of our data (4-year overall survival rate of 69.1%) showed close results to the retro-EMBRACE study (5-year overall survival rate of 65%) and Narayan *et al.* (5-year overall survival rate of 65%) [7, 19].

Consequently, our analysis showed that 4-year local control, progression-free survival and overall survival rates were 89.5%, 74.9% and 69.1%, respectively. The prognostic factors for local control were non-SCCA histology, advanced stage, cumulative dose > 84 Gy and OTT > 56 days. The prognostic factors for overall survival were non-SCCA histology, advanced stage, presented LN and longer OTT. To increase the cumulative dose by hybrid IC/IS technique and reduce OTT by strictly ‘twice a week’ schedule will be our plan to improve our treatment outcomes in the future.

## CONCLUSION

The 4-year overall survival from our experiences was 69.1% closed to the retro-EMBRACE study. Non-SCCA histology, advanced stage, presented LN and longer OTT effected the overall survival in multivariate analysis.

## CONFLICT OF INTEREST

The authors declare they have no conflicts of interest.
